# Modeling of SGLT1 in Reconstituted Systems Reveals Apparent Ion-Dependencies of Glucose Uptake and Strengthens the Notion of Water-Permeable Apo States

**DOI:** 10.3389/fphys.2022.874472

**Published:** 2022-06-15

**Authors:** Thomas Barta, Walter Sandtner, Johann Wachlmayr, Christof Hannesschlaeger, Andrea Ebert, Armin Speletz, Andreas Horner

**Affiliations:** ^1^ Department of Molecular Biophysics and Membrane Biophysics, Institute of Biophysics, Johannes Kepler University Linz, Linz, Austria; ^2^ Center of Physiology and Pharmacology, Institute of Pharmacology and the Gaston H. Glock Research Laboratories for Exploratory Drug Development, Medical University of Vienna, Vienna, Austria; ^3^ Department of Analytical Environmental Chemistry, Helmholtz Centre for Environmental Research—UFZ, Leipzig, Germany

**Keywords:** human sodium glucose co-transporter, water-permeable apo states, solute carrier, glucose uptake, lipid vesicles, mathematical modeling, passive membrane permeabilities

## Abstract

The reconstitution of secondary active transporters into liposomes shed light on their molecular transport mechanism. The latter are either symporters, antiporters or exchangers, which use the energy contained in the electrochemical gradient of ions to fuel concentrative uptake of their cognate substrate. In liposomal preparations, these gradients can be set by the experimenter. However, due to passive diffusion of the ions and solutes through the membrane, the gradients are not stable and little is known on the time course by which they dissipate and how the presence of a transporter affects this process. Gradient dissipation can also generate a transmembrane potential (V_M_). Because it is the effective ion gradient, which together with V_M_ fuels concentrative uptake, knowledge on how these parameters change within the time frame of the conducted experiment is key to understanding experimental outcomes. Here, we addressed this problem by resorting to a modelling approach. To this end, we mathematically modeled the liposome in the assumed presence and absence of the sodium glucose transporter 1 (SGLT1). We show that 1) the model can prevent us from reaching erroneous conclusions on the driving forces of substrate uptake and we 2) demonstrate utility of the model in the assignment of the states of SGLT1, which harbor a water channel.

## 1 Introduction

Solute carriers (SLCs) serve as gatekeepers for vital physiological functions, including cellular uptake of minerals, vitamins, and nutrients as well as removal of metabolites from the interior of cells (Hediger et al., 2004; Omote et al., 2006; Sano et al., 2020). Mutations in the genes encoding SLC proteins are linked to a plethora of diseases, such as amyotrophic lateral sclerosis [prevalence in the United States in 2016; 0.52% (Mehta et al., 2021)], Alzheimer’s disease [prevalence in Europe; 5.05% (Niu et al., 2017)], and schizophrenia [prevalence in 46 countries worldwide; 0.46% (Saha et al., 2005)]. Extrapolated to the world population of 2021, these three conditions alone amount to as many as 470 million annual patients. In addition, SLC’s like the sodium glucose transporter 1 (SGLT1) play a pivotal role in metabolism, i.e., glucose uptake. Although SLCs have been studied for many years using biophysical and biochemical approaches as for example electrophysiology and radio-ligand uptake experiments, nevertheless, there is a great deal that we still don’t know about their true conformations whilst working against concentration gradients. Hence, there is an urgent need to employ novel approaches so as to gain insight into how SLCs operate.

SLCs undergo long range conformational changes between inward- and outward-open states. As a consequence, the substrate can either bind to the extra- or the intracellular side of the protein but not to both sides simultaneously. For this reason, all SLCs operate within the conceptual framework of the alternating access model (Jardetzky, 1966). Many of them can harvest the energy contained in ion gradients to drive uphill transport of their substrate against an opposing gradient. Others work as exchangers or facilitate passive diffusion of polar solutes. In addition to the ion and substrate gradients, the membrane potential (V_M_) also impinges on transporter function. As intimated above, solute carrier function is classically explored by monitoring uptake of radiolabeled substrate into a cell in which the transporter is expressed. However, substrate accumulation can also be measured in an artificial vesicle system relying on radioactivity (Billesbolle et al., 2016) or fluorescence (Erokhova et al., 2016). Whereas the V_M_ across cellular membranes is largely stable, it is a dynamic variable in artificial vesicle systems. This is because in cells, the ion gradients are maintained by pumps so that they do not dissipate on passive diffusion and transport by SLCs, which is not the case in proteoliposomes. Hence, disregarding V_M_ in *in vitro* experiments may lead to erroneous conclusion on the driving forces of substrate uptake by SLCs.

Of the SLC group of proteins, SGLT1 is one of the best studied representatives. This transporter is expressed at high levels on the microvilli of the brush border membrane of enterocytes within the alimentary canal (Hwang et al., 1991). In addition to nutrients, the small intestine also absorbs approximately 8 L of fluid daily. Because aquaporines are absent in enterocytes, it was speculated that SGLT1 acts as conduit for water. Primary experimental evidence for the existence of a water channel passing through the transporter came from *in vitro* experiments utilizing reconstituted human SGLT1 and from experiments conducted on this protein while expressed in cell monolayers. Per second, the number of transported water molecules (10^9^) exceeded by far the glucose turnover number of SGLT1, which was only in the order of a few hundred molecules (Erokhova et al., 2016). These results led to the postulation that perhaps additional SLCs (Erokhova et al., 2016; Zeuthen et al., 2016) might also be important in the regulation of transcellular water flux. Molecular dynamics (MD) simulations confirmed the passive water transport capability of SGLT1 in its inward-facing state (Li et al., 2013; Adelman et al., 2014). Moreover, a continuous water channel passing within the sugar-binding site was identified in the bacterial homologue vSGLT (Li et al., 2013; Adelman et al., 2014). Extensive simulations carried out on a variety of transporters superfamilies concluded that water-conducting states likely represent a universal phenomenon in membrane transporters, including those belonging to the solute sodium symporter; solute carrier 1; major facilitator superfamily; nucleobase-cationsymport-1; and ATP binding cassette families. It was speculated that this phenomenon is a consequence of transporter reliance on large-scale motions (Li et al., 2013). Experimental results using human SGLT1 (Erokhova et al., 2016; Zeuthen et al., 2016) indicated a shared pathway of water with glucose but not with Na^+^. However, it is still unknown which states in the transport cycle are permeable for water and which are not.

In the present study, we built a mathematical model of SGLT1 incorporated into an artificial membrane (i.e., unilamellar vesicles). To model the flux of substrate and co-substrate through the transporter, we utilized a previously published kinetic model of human SGLT1 (Wright et al., 2011). Importantly, our approach also accounted for passive diffusion of ions and solutes through the vesicular membrane. We provided predictions regarding the intraluminal concentrations of the ions and solutes, vesicle volume (through osmotic effects), and the transmembrane potential change on (experimental) perturbation (e.g., challenge with the substrate or osmolytes). Another output of the computation were the state occupancies of SGLT1, which depended on the above-mentioned parameters (e.g., V_M_ and ion concentrations). We emulated two types of experiments: 1) radio-ligand uptake experiments and 2) stopped flow experiments of the kind that allow for measuring water flux. With these two approaches we demonstrated the utility of our equations in the interpretation of experimental outcomes.

## 2 Results

### 2.1 Parameterizing a Virtual Model for a Unilamellar Vesicle

**FIGURE 1:**
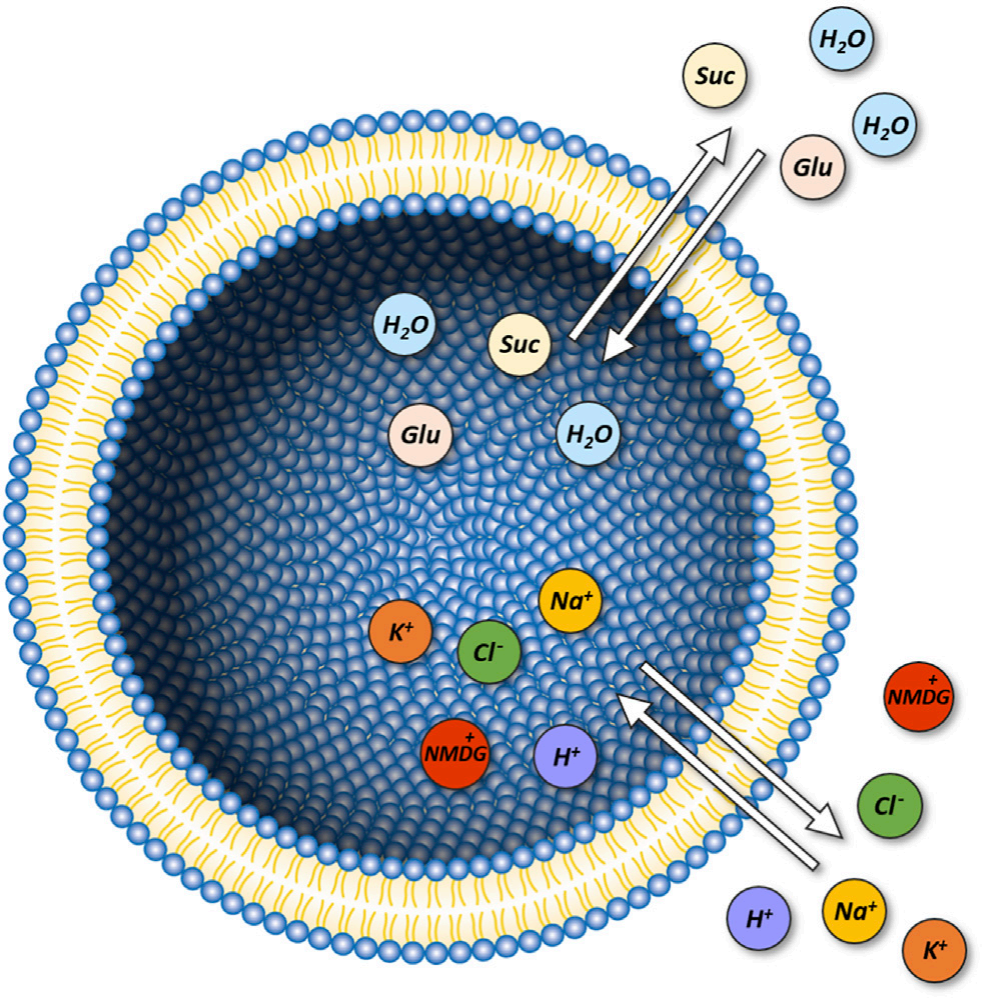
Model of a unilamellar vesicle. Initial  NaCl , KCl , buffer and proton concentrations inside and outside the vesicle can be set to user-defined values. An osmotic solute (e.g., sucrose) can be applied to the extra-vesicular side to simulate water flux experiments utilizing a stopped-flow device. The lipids comprising the membrane are shown with their headgroups in blue and their acyl chains in yellow. The arrows indicate passive permeation of $H^{+}$ , $Na^{+}$ , NMDG^+^, $Cl^{-}$ , and $K^{+}$ . In the model we also accounted for passive flux of water (light blue circles), glucose, sucrose and NMDG_0_ (beige circles).

Displayed in Figure 1 is a schematic representation of our model system (i.e., unilamellar vesicle). This system is comprised of a lipid bilayer, which separates the intra-vesicular space from the surrounding bath solution. In the model, the initial concentrations of the ions and the sugars (i.e., glucose and sucrose) on the opposing sides of the membrane can be set to user-defined values. On start of the simulation, the system equilibrates. Depending on the chosen initial concentrations of the ions and the sugars the following changes can ensue: 1) changes in vesicular volume, if the osmotic concentrations of the intra-and extra-vesicular solutions differ, 2) changes in the concentrations of the intra-vesicular ions and sugars due to passive diffusion of these molecules through the membrane and 3) changes in membrane potential, caused by positive/negative net-charge leaving or entering the vesicle. Indicated in the diagram are the passive fluxes of the ions [i.e., Na^+^, K^+^, H^+^, Cl^−^, and NMDG^+^ (N-Methyl-D-glucamin)], water, and glucose/sucrose. In the model, we parameterized these fluxes, with membrane permeabilities (P_M_ values) obtained from the literature. In the case of NMDG^+^ we determined this value experimentally (see Section 4). For simplicity, we assumed that the extra-vesicular concentrations of all ions and sugars remain constant. This was justified by the fact that in the experiments, which we simulated, the extra-vesicular volume exceeded the combined volume within the vesicles by more than two orders of magnitude. For this reason, depletion of the solutes in the bath solution was considered negligible. A comprehensive description of the model can be found in Section 4.

### 2.2 Passive Ion Permeation Through the Vesicular Membrane can Generate a Membrane Potential (V_M_)

Ions differ in their ability to passively permeate through membranes (Hannesschlaeger et al., 2019a). For instance, anions like Cl^−^ can cross membranes more readily than cations such as Na^+^. This is because of the dipole potential, which exists within the membrane and which repels positive charges. If the magnitudes of the passive fluxes of cations and anions differ and if for these ions a transmembrane gradient exists, a net flux of charge through the membrane ensues. The same is true, if the different species of cations and anions, present in the assay, permeate through the membrane with differing velocities due to differences in their ionic radii (e.g., Na^+^ versus K^+^ or Na^+^ versus NMDG^+^). The resulting net flux of charge gives rise to a change in the membrane potential (V_M_). In turn, the electrical field generated by V_M_ impinges on the flux of the charged particles.

**FIGURE 2:**
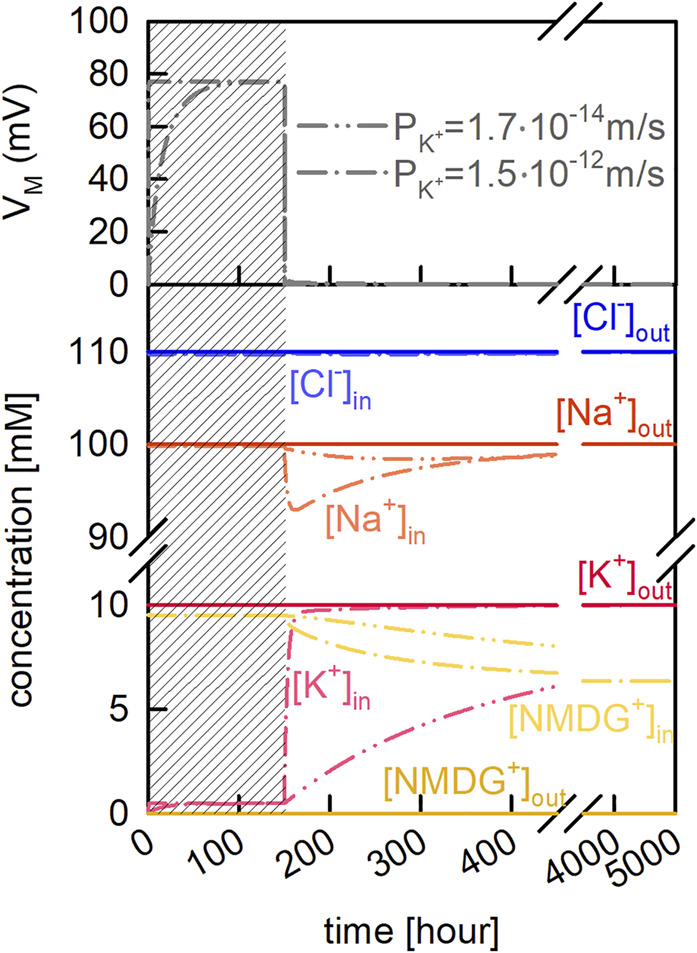
Passive ion permeation through the vesicular membrane. Vesicle assay kinetics simulated with two different P_M_ values for potassium (taken from Table 2 of Paula et al. [Paula et al., 1996)]. Dash-dot-dotted lines and dash-dotted lines show simulations in which $P_{K^{+}}$ was set to $1.7\cdot 10^{-14}m/s$ , and $1.5\cdot 10^{-12}m/s$ , respectively. The initial extraluminal concentrations were 100 mM NaCl, 20 mM HEPES, and 10 mM KCl. The initial intraluminal concentrations were 100 mM NaCl, 20 mM HEPES, and 10 mM NMDG^+^Cl^−^. The pH on both sides of the membrane was 7.5. Upper panel. Time course of the change in membrane potential ( $V_{M})$ . On start of the simulations, K^+^ was the only permeable solute (grey shaded area). After 150 h, all other ions ( $H^{+}$ , $Na^{+}$ , $NMDG^{+}$ , and $Cl^{-}$ ) were allowed to permeate. Lower panel. Evolution of all intra- (bright colors) and extraluminal (dark color) ion concentrations (except for H^+^) over time.

We used our model of the vesicle to better understand how passive ion fluxes through the membrane affect V_M_ and the intra-vesicular ion concentrations. In the example depicted in Figure 2 we assumed that the Na^+^ and Cl^−^ concentrations were the same on both sides of the membrane (i.e., 100 mM NaCl). We further assumed the additional presence of 10 mM K^+^Cl^−^ and 10 mM NMDG^+^Cl^−^ on the extra- and intraluminal side, respectively. We stress that in the chosen example, the osmotic concentrations of the inner and outer solution were kept equal. In a first equilibration step we only allowed for passive diffusion of K^+^ (grey shaded area in Figure 2). In the upper panel of Figure 2 we show V_M_ as a function of time for two different reported P_M_ values of K^+^. On equilibration, about 220 K^+^ ions entered the lumen of the vesicle, upon which V_M_ adopted a value of approximately 80 mV. At this V_M_ the electric force equaled the force exerted by the chemical potential of K^+^, which is why the K^+^ flux seized. After equilibrium was reached (i.e., 150 h) we let all other ion-species permeate. As seen, this gave rise to anew change in V_M_ and the intracellular ion concentrations (lower panel of Figure 2). However, in contrast to the changes which had ensued when K^+^ was the only permeating ion, the changes on permeation of all ions were transient. This was because with all ions permeating their gradients eventually dissipated. This is evident on inspection of Figure 2, where we show that the final intra-vesicular ion concentrations matched exactly the concentrations on the extra-vesicular side. In the absence of ion gradients, V_M_ was zero and no osmotic force was exerted onto the membrane. The transient changes in V_M_ and the intracellular ion concentrations occurred before the system had reached equilibrium. However, the extent and time course of these changes depended on the velocities by which the different ion species traveled through the membrane. This is evident from the two plotted curves in Figure 2, which show that the changes in vesicle parameters were highly dependent on which of the two reported membrane permeabilities for K^+^ were used in the simulation.

### 2.3 The Membrane Potential Across Vesicular Membranes Depends on the Initial Ion Concentrations at the Intra-and Extra-Vesicular Side

Secondary active transporters use the energy contained in the electrochemical gradient of ions to fuel concentrative uptake of their cognate substrate (Burtscher et al., 2019). Hence, assessment of their function relies on experimental systems, which allow for adjustment of the transmembrane ion gradients. In vesicles these gradients can be set by the experimenter. However, as shown before, in the vesicle these gradients and the membrane potential are expected to change in a time dependent manner, due to passive diffusion of ions through the membrane. This can complicate the interpretation of experimental outcomes. We demonstrate this with an example.

Let’s assume we believe that a candidate Na^+^ symporter antiports K^+^ in the return step from the substrate-free inward- to the substrate-free outward-facing conformation and that it can thereby tap the energy contained in the electrochemical gradient of K^+^. This, for instance, was shown to be the case for the serotonin transporter (SERT-SLC6A4) and for transporters in the SLC1 family (glutamate transporters) (Rudnick and Sandtner, 2019; Wang, et al., 2020). We could test this by preparing vesicles, which contain K^+^ or the inert cation NMDG^+^ instead. Let’s further assume that we find the concentrative power of the secondary active transporter, which we reconstituted into these vesicles, higher in the presence than in the absence of intra-vesicular K^+^. Is it now safe to conclude that this transporter antiports K^+^?

**FIGURE 3:**
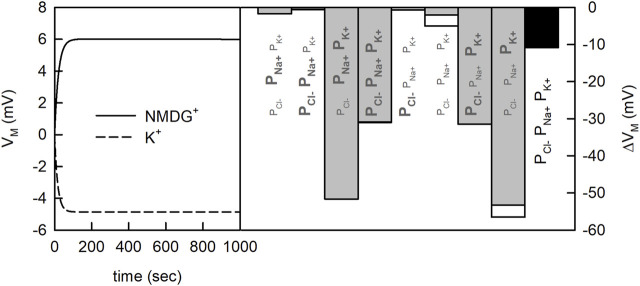
Membrane potential generated by the net flux of charged particles. Vesicle assay kinetics simulated for two different experimental conditions. At both conditions, 200 mM NaCl was present outside the vesicle. Inside the vesicle there was either 200 mM KCl or 200 mM NMDG^+^Cl^−^. In the simulation 20 mM HEPES, was present inside and outside the vesicle and the pH was set to 7.5. Left panel Time course of the change in membrane potential $\left(V_{M}\right)$ for a set of passive membrane permeabilities (i.e., $P_{Cl^{-}}=5\cdot 10^{-13}m/s$ , $P_{K^{+}}=3\cdot 10^{-13}m/s$ , $P_{Na^{+}}=1.5\cdot 10^{-13}m/s$ ). Right panel Differences in transmembrane potentials between the two different experimental conditions for permutations of maximal and minimal P_M_ values of Cl^−^, Na^+^ and K^+^ reported in the literature (grey bars) (bold values in Table 1) as well as our exemplary set (black bar). Minimal P_M_ values are indicated using smaller font size, maximal P_M_ values using bold letters. The effect of $P_{NMDG_{0}}=1\cdot 10^{-11}m/s$ is shown with empty white bars. $\Delta V_{M}$ was defined as the maximal difference between *V_M,K^+^_* − *V_M,NMDG^+^_*.

The issue, here, is that the concentrative power of a secondary active transporter can also depend on V_M_, if the transporter under scrutiny is electrogenic (i.e., one or more net charges are transported through the membrane in each transport cycle). The question, therefore, is whether the two tested experimental conditions can give rise to differing V_M_s. In Figure 3 (left panel) we show the time course of the V_M_ change on equilibration for the two different conditions. The chosen P_M_ values for Na^+^, Cl^−^, and K^+^ are indicated in the figure (black bar). We simulated the evolution of V_M_ for 1,000 s. As seen, on start of the simulation V_M_ rapidly diverged from zero. More importantly, the V_M_ values for the two conditions differed by about 10 mV after a few minutes. Given this result we can no longer be sure that the increase in concentrative power, which we observed in the experiment, is supportive of K^+^ antiport. Arguably, this increase could also have been caused by the difference in V_M_. Admittedly, the divergence in the V_M_ values in the shown simulations was conditioned on the chosen P_M_ values for the ions. We obtained the values for Na^+^, Cl^−^ and K^+^ from the literature. Yet, the spread in the reported values for these ions is considerable (see Table 1). For NMDG^+^ no experimentally determined P_M_ value was available. Because of its large size this cation was considered impermeable (Dhoke et al., 2005; Elustondo et al., 2013; Reuss, 1979). However, what has been frequently overlooked is that NMDG can exist in an uncharged form (NMDG_0_; pKa = 8.8). The latter is expected to diffuse through the membrane at a larger rate. For this reason, we determined the P_M_ value for NMDG_0_ experimentally (see Section 4). In Figure 3 (right panel) we show the predicted difference (∆V_M_) between the two experimental conditions when using different combinations of reported P_M_ values. Each bar shows the result of a simulation in which we did or did not account for diffusion of NMDG_0_. It is evident that only for two of the chosen combinations this made a small difference (white bars indicate results obtained when NMDG_0_ was assumed to permeate). In summary we conclude from the data depicted in Figure 3, that 1) the magnitude of ∆V_M_ depends on the chosen combination of P_M_ values, 2) that V_M_ is always more negative in the presence than in the absence of an outwardly directed K^+^ gradient and 3) that the influence of the uncharged form of NMDG on V_M_ is negligible.

**TABLE 1:** Literature values for passive membrane permeabilities of Cl^−^, K^+^, and Na^+^. Indicated are the lipid composition and temperature for which the value was estimated and the method employed for the measurement. Bold values indicate for each ion the values for $P_{max}$ and $P_{min}$ chosen in the simulations.

Type of Ion	Value [10^−13^ m/s]	Lipid composition	Method	T [°C]	References
Cl^−^	1,000	POPC	pH sensitive fluorophore in vesicles	25	Megens et al. (2014)
	0.1–100	egg lecithin	—	—	Kanehisa & Tsong, (2002)
	40	Egg yolk phosphotidylcholine	pH sensitive vesicle experiments	23	Nozaki & Tanford, (1981)
	**1.5**–11.3	egg phosphatidylcholine	radioactive labelling	3.2–20	Toyoshima & Thompson, (1975)
	7.6	Hen egg lecithin	radioactive labelling	24	Mimms, Zampighi, Nozaki, Tanford, & Reynolds, (1981)
	**5.5**	egg lecithin	radioactive labelling	4	Hauser, Phillips, & Stubbs, (1972)
	0.65	PS	radioactive labelling	36	Papahadjopoulos, Nir, & Oki, (1972)
	0.37	PS-Chol	radioactive labelling	36	Papahadjopoulos et al. (1972)
K^+^	**0.17**–**15**	phosphatidylcholines with monounsaturated fatty acids composed of 14–24 carbon atoms	Electrodes	30	Paula et al. (1996)
	0.091	PS	radioactive labelling	36	Papahadjopoulos et al. (1972)
	0.033	4% phosphatidic acid-phosphatidyl choline	radioactive labelling	37	Johnson & Bangham, (1969)
	0.01	POPC	pH sensitive measurement	22	Shen et al. (2020)
	0.0047	PS-Chol	radioactive labelling	36	Papahadjopoulos et al. (1972)
Na^+^	1,000	POPC	pH sensitive fluorophore in vesicles	25	Megens et al. (2014)
	3–**27**	egg phosphatidylcholine in decane	electrical conductance and pH electrode techniques	24	John Gutknecht & Anne Walter, (1981a)
	0.15	PG	radioactive labelling	36	Papahadjopoulos et al. (1972)
	0.15	Egg yolk phosphotidylcholine	pH sensitive vesicle experiments	23	Nozaki & Tanford, (1981)
	0.1	egg lecithin	—	—	Kanehisa & Tsong, (2002)
	0.095	Hen egg lecithin	radioactive labelling	24	Mimms et al. (1981)
	0.05	Egg yolk phosphotidylcholine	pH sensitive vesicle experiments	23	Nozaki & Tanford, (1981)
	0.021	egg lecithin	radioactive labelling	25	Brunner et al. (1980)
	0.02	PG-Chol	radioactive labelling	36	Papahadjopoulos et al. (1972)
	0.016	PS	radioactive labelling	36	Papahadjopoulos et al. (1972)
	0.011	PA-PC	radioactive labelling	36	Papahadjopoulos et al. (1972)
	0.01	PA-PC-Chol	radioactive labelling	36	Papahadjopoulos et al. (1972)
	0.005	PS-Chol	radioactive labelling	36	Papahadjopoulos et al. (1972)
	**0.0012**	egg lecithin	radioactive labelling	4	Hauser et al. (1972)

### 2.4 Model of a Unilamellar Vesicle Harboring SGLT1

To predict substrate uptake by the transporter into the vesicle, we incorporated SGLT1 into our model system. To this end, we used a previously published kinetic model of SGLT1 (see Figure 8 in Section 4). The established stoichiometry of SGLT1 is that two Na^+^ ions and one glucose molecule are transported through the membrane in each transport cycle. Reconstituted proteins are usually randomly oriented. We accounted for this in the model by assuming that one half of the SGLT1 units was oriented in the inside-out and the other half in the inside-in configuration.

It is possible with the kinetic model to calculate the flux of glucose and Na^+^ through SGLT1. However, the magnitudes of these fluxes depend on the intra-and extraluminal Na^+^ and glucose concentrations and on V_M_. In the bare vesicle, the extents and time courses by which these vesicle parameters changed were conditioned on the rates by which the different ion species passively permeated. With SGLT1 incorporated into the vesicle, the situation is different. For instance, in the presence of SGLT1, a set Na^+^ gradient is expected to dissipate faster because of the additional Na^+^ ions, which can enter the vesicle via the transporter. Accordingly, with SGLT1 present, the evolution of the vesicle parameters over time ought to be different from that in its absence.

**FIGURE 4:**
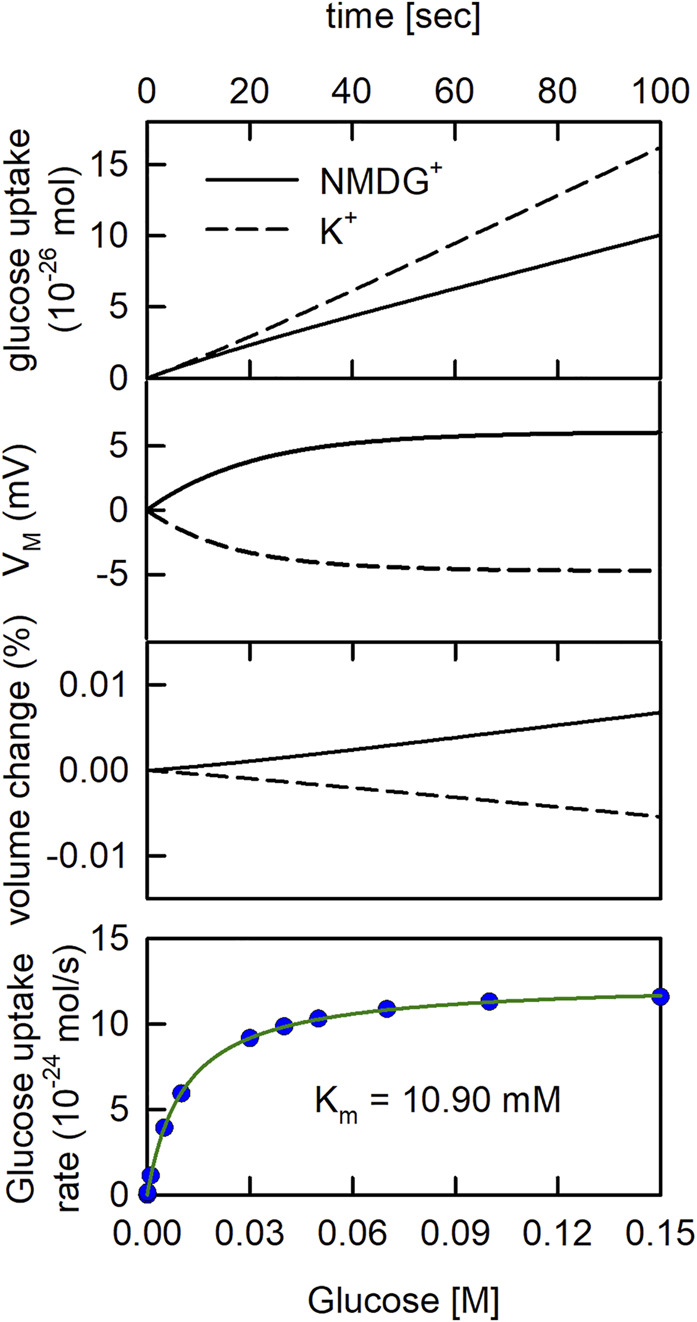
The glucose uptake rate of SGLT1 is larger in the presence than in the absence of a K^+^ gradient. Upper. Glucose uptake by SGLT1. In the simulation 1 µM glucose was applied to the extraluminal side of the vesicle. In the *in-silico* experiment, we assumed that on average 4.3 monomers were inserted into the vesicle with half of them oriented outside-in (Erokhova et al., 2016). The extraluminal concentration of 200 mM NaCl was osmotically balanced with either 200 mM KCl or 200 mM NMDG^+^Cl^−^ in the vesicle lumen. The rate of glucose uptake was higher in the presence of a K^+^ gradient (dashed line) than in its absence (solid line). Upper middle panel Time course of V_M_ change. V_M_ was more negative in the presence of the K^+^ gradient (dashed line) than in its absence (solid line). Lower middle panel Time course of vesicle volume change. The volume of the vesicle slightly increased and decreased when 200 mM NMDG^+^(solid line) and 200 mM K^+^ (dashed line) were initially contained in the vesicle, respectively. Lower panel Simulated glucose uptake as a function of glucose concentration (blue dots). The synthetic data were fit to the Michaelis Menten equation (green curve): $Glu\cos e\;uptake\;rate=\left(V_{\max}\cdot \left[S\right]\right)/\left(K_{m}+\left[S\right]\right)$ . The $K_{m^{-}}$ and the $V_{\max}$ value estimated by the fit were 10.90 mM and $V_{\max}$ of 1.22 ± 0.006 · 10^−23^ mol/s (simulation conditions: 150 mM NaCl, 20 mM MOPS, and pH 7.5 with 4.3 hSGLT1’s per vesicle), respectively.

In Figure 4 we revisited the question, which we posed above. That is: is it safe to conclude that a solute carrier antiports K^+^ if we find its concentrative power increased in the presence of a transmembrane K^+^ gradient? To tackle this question, we conducted simulations in the assumed presence and absence of a K^+^ gradient, respectively. However, in contrast to the simulation shown above (Figure 3) we now assumed that SGLT1 was inserted into the vesicular membrane. The reaction scheme of SGLT1 does not specify K^+^ binding to the transporter. Accordingly, if in the simulations the presence of a K^+^ gradient gives rise to elevated glucose uptake by SGLT1 we know that this cannot be due to K^+^ antiport. In Figure 4 (upper panel), we simulated glucose uptake into the vesicle for a period of 100 s on addition of 1 µM glucose to the extra-vesicular solution. We note that 1 µM is within the range of concentrations, which are typically employed in radioligand uptake assays. As seen, in the presence of the K^+^ gradient, uptake of glucose into the vesicle was increased. In Figure 4 (upper middle panel) we plotted the evolution of V_M_ over time for the two experimental conditions. The plot confirmed that the observed increase in glucose uptake in the presence of the K^+^ gradient had been a corollary of the more negative V_M_, which had built up at this condition. In the lower middle panel of Figure 4 we show the time dependent change in vesicular volume in percent. We also simulated glucose uptake as a function of the extra-vesicular glucose concentration (lower panel of Figure 4). These data were fit to the Michaelis Menten equation. The K_m_ and the V_max_ values for glucose uptake estimated by the fit were 10.90 mM and 1.22 ± 0.006 10^−23^ mol/s, respectively.

**FIGURE 5:**
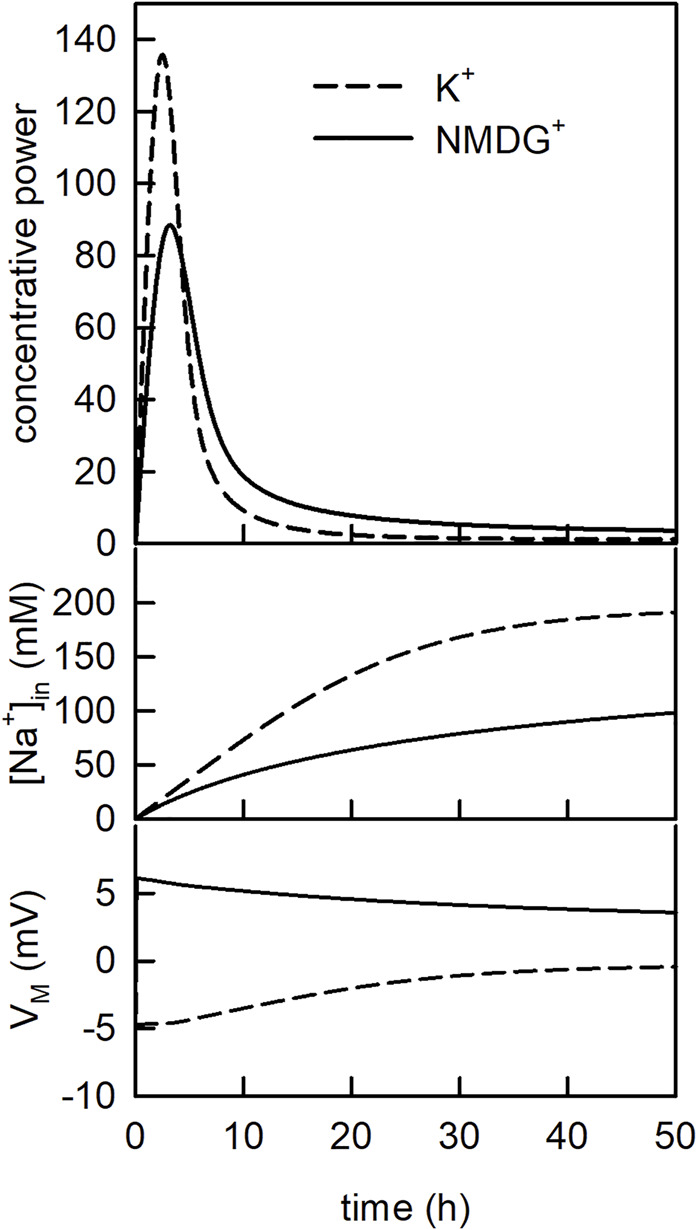
The concentrative power of SGLT1 changes over time. Upper panel Evolution of the concentrative power of SGLT1 over time. In the simulation 1 µM glucose was applied for 50 h. The dashed line and the solid line show the concentrative power ([Glu]_in_/[Glu]_out_) in the presence and in the absence of the K^+^ gradient, respectively. Middle panel Time course of the intraluminal sodium concentration [Na^+^]_in_ for the two conditions. Lower panel Time course of the membrane voltage ( $V_{M}$ ) for the two conditions.

In Figure 5 (upper panel) we plotted the concentrative power of SGLT1 (i.e., [Glu]_in_/[Glu]_out_) as a function of time in the assumed presence (dashed line) and absence (solid line) of a K^+^ gradient. In the simulation we applied 1 µM glucose to the extraluminal side of the vesicle for 50 h. As seen, at both conditions, the concentrative power first increased and then decreased. The decrease occurred on dissipation of the Na^+^ gradient (Figure 5 **-**middle panel). This transient change in concentrative power is a frequently observed phenomenon if radio-ligand uptake is monitored over an extended period of time (i.e., overshoot phenomenon) (Heinz and Weinstein, 1984). Most notably, however, we found the concentrative power of SGLT1 increased when the K^+^ gradient was present. Since SGLT1 did not interact with K^+^, this was also a consequence of the differing V_M_s between the two different experimental conditions (Figure 5 **-**lower panel).

### 2.5 State Occupancies Depend on the Intra- and Extra Luminal Buffer Conditions

Our model system can also be used to address more sophisticated questions. We demonstrate this with an example. We recently showed that SGLT1 is highly permeable for water (Erokhova et al., 2016). We also found that the conductivity of SGLT1 for water was not contingent on the presence of the co-substrate (i.e., Na^+^), because when K^+^ was used as the substituting ion, the water flux remained essentially unchanged. Addition of 20 mM glucose, on the other hand, decreased water transport through SGLT1 by approx. 17%. However, it remained unclear, which conformation visited during glucose transport had or had not been water permeable. Here, we tackled this question with our model.

In the actual measurement of water flux through SGLT1, vesicles which contained this transporter (proteoliposomes) were subjected to a hyperosmotic buffer in a stopped-flow device. Application of the hyperosmotic buffer led to shrinkage of the vesicles, which was monitored by measuring the scattered light intensities at 90° (Hoomann et al., 2013; Hannesschlager et al., 2018). These data allowed for calculating the unitary water permeability through SGLT1 (Horner et al., 2015). To emulate stopped-flow experiments with our mathematical model we first let the proteoliposomes (PL) equilibrate. We then applied a step change of sucrose/glucose with the characteristic time constant of the dead time of our stopped-flow device (*τ* = 2 ms). To gain a deeper understanding of our published data on water flux through SGTL1 (Erokhova et al., 2016) we simulated three experimentally tested conditions: 1) PLs reconstituted in 150 mM KCl buffer and subjected to a 150 mOsm sucrose gradient, 2) PLs reconstituted in 150 mM NaCl buffer and subjected to a 150 mOsm sucrose gradient and 3) PLs reconstituted in 150 mM NaCl buffer and subjected to a hyperosmotic gradient of 150 mM sucrose and 20 mM glucose.

**FIGURE 6:**
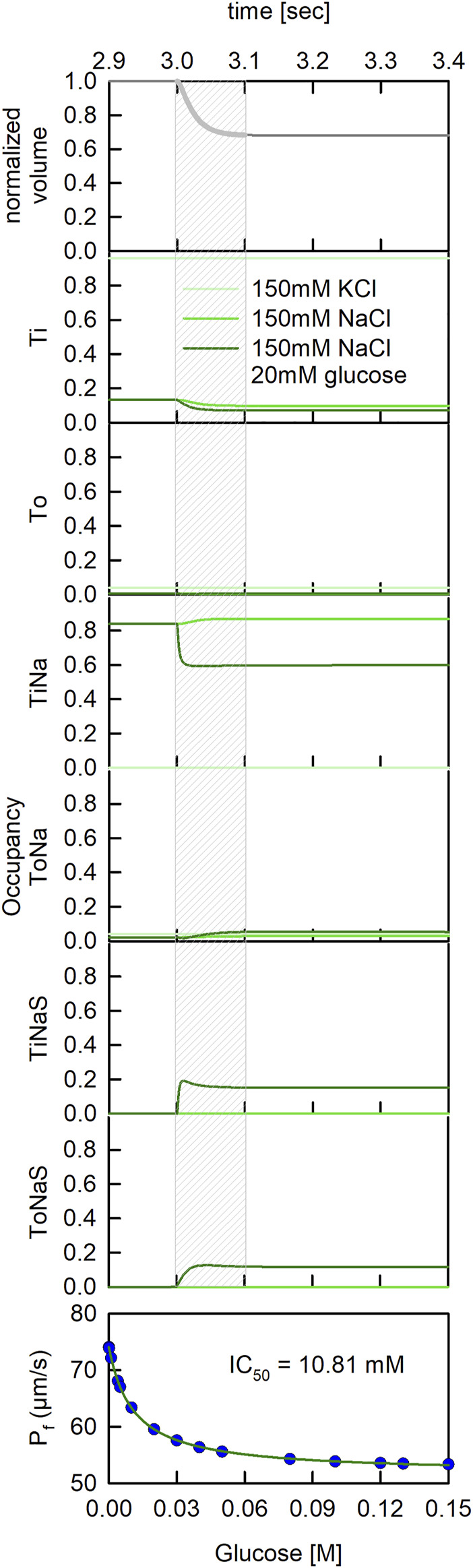
Dynamic changes of state occupancies in a stopped flow experiment. State occupancies of SGLT1 before and after application of a hyperosmotic gradient (at time *t* = 3 s). We simulated three different experimental conditions: (light green line) PLs reconstituted in 150 mM KCl buffer and subjected to a 150 mOsm sucrose gradient, (green line) PLs reconstituted in 150 mM NaCl buffer and subjected to a 150 mOsm sucrose gradient and (dark green line) PLs reconstituted in 150 mM NaCl buffer and subjected to a hyperosmotic gradient of 150 mM sucrose and 20 mM glucose. The grey shaded area indicates the time period during which the vesicles shrunk (see uppermost panel). It was during this period that the scattered light intensities changed and that we obtained information on water flux through SGLT1.

In our model, application of the hyperosmotic buffer led to a spontaneous decrease of vesicle volume (Figure 6-upper panel). The volume settled at about two-thirds of its original value, 100 ms after application of the osmolyte. The decrease in volume was accompanied by concentration changes of the solutes in the vesicle (data not shown). An additional output of our model are the state occupancies of SGLT1, which because they depended on the vesicle parameters, also changed on application of the osmolyte. In Figure 6 we plotted the state occupancies of all states specified in the reaction scheme of SGLT1. The equilibrated proteoliposomes were challenged with 150 mM sucrose 3 seconds after start of the simulation. The three curves in each panel are the state occupancies for the three experimentally tested conditions. In the lower panel of Figure 6 we also plotted the inhibitory effect of glucose on water flux as a function of the applied glucose concentration. These data were fit to an inhibition curve. The fit yielded an IC_50_ value of 10.81 mM. This value agreed well with the *K*_*m*_ for glucose uptake (cf. Figure 4 -lower panel and Figure 6 -lower panel).

**FIGURE 7:**
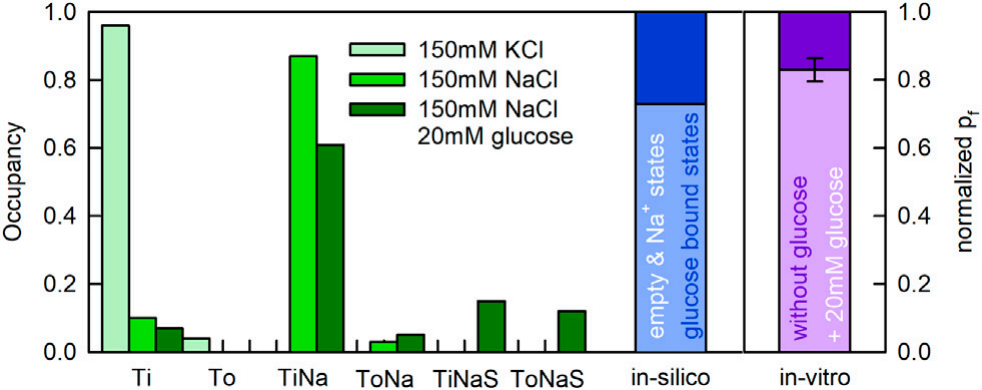
State occupancies of SGLT1 during water flux though the transporter. Upper panel To and Ti represent the outward and inward facing apo-conformations, respectively (Figure 8). ToNa, TiNa, ToNaS, and TiNaS denote the sodium and the sodium/glucose bound states. State occupancies were averaged over the first 100 ms after application of the osmolyte (Figure 6). We used the same color code as in Figure 6 to indicate the different experimental conditions. The bar in blue shows the summed fraction of the substrate bound states (dark blue) and, the summed fraction of the substrate free states (light blue). The difference between the light purple and dark purple bar shows the decreased unitary water permeability measured in the presence of 20 mM glucose. Lower panel Simulated water permeability of SGLT1 (assuming water impermeable glucose states) as a function of the glucose concentration (blue dots). The green curve denotes the fit of an inhibition curve to the data: $P_{f}=b+\left(a-\left(a\cdot \left[S\right]\right)\right)/\left(\left[S\right]+IC_{50}\right)$ The extracted $IC_{50}$ value was 10.81 mM (simulation conditions: 150 mM NaCl, 20 mM MOPS, and pH 7.5 with 4.3 hSGLT1’s per vesicle +150 mM sucrose outside the vesicle).

Displayed in Figure 7 are the state occupancies averaged over the period during which the proteoliposomes shrunk (i.e., the first 100 ms following the application of sucrose). This is the period during which we had obtained information on the water flux through SGLT1. As seen, in the presence of 150 mM KCl the transporter mostly dwelled in the apo-inward facing conformation (Ti—96% occupancy) (see Figure 7). In contrast, in the presence of 150 mM NaCl, SGLT1 adopted the sodium bound inward facing conformation (TiNa—87% occupancy) and the Ti state (10% occupancy). In the additional presence of 20 mM glucose, the transporter also visited substrate bound states (i.e., TiNaS—12% occupancy and ToNaS—15% occupancy).

We note that the summed fraction of glucose bound states amounted to 27% when 20 mM glucose was present. Because water flux through SGLT1 was reduced by approximately 17% at the same condition, it is tempting to speculate that it were the substrate-free states which conducted water and the substrate bound states which were water impermeable. The blue bar in Figure 7 shows the summed fraction of the substrate-free states (in light blue) and the summed fraction of the substrate bound states (in dark blue) in the presence of 20 mM glucose. The bar in purple summarizes our recent experimental findings (i.e., water flux in the presence of glucose normalized to the water flux measured in its absence). The occupancies of the substrate bound states and the loss in water flux were in reasonable agreement.

## 3 Discussion

Here, we present an *in-silico* model, which allows to predict the time courses of solute concentration changes within the confinement of a unilamellar vesicle, both in the absence and presence of a glucose transporter (SGLT1). The model accounts for passive diffusion of ions, sucrose, glucose, and water through the vesicular membrane and for solute flux through the transporter (i.e., Na^+^ and glucose). In addition, we also incorporated state-dependent water flux through SGLT1. In the model, the solutes and solvent, which entered or left the vesicle gave rise to changes in the intra-vesicular solute concentrations, vesicle volume and transmembrane potential (V_M_). These parameters in turn impinged on the operation of the embedded transporter. The model can emulate a wide range of experiments and it can therefore be used to predict and interpret experimental outcomes. In addition, it can help find optimal experimental conditions. That is: it can 1) assist in ruling out initial conditions, which would lead to vesicle rupture due to excessive volume increase and/or large membrane potential, and 2) provide estimates for the expected signal change to help chose the measurement strategy, which has the highest chance of success. In the present study, we simulated radioligand uptake and stopped-flow experiments, of the kind used to measure water flux through proteoliposomes.

**FIGURE 8:**
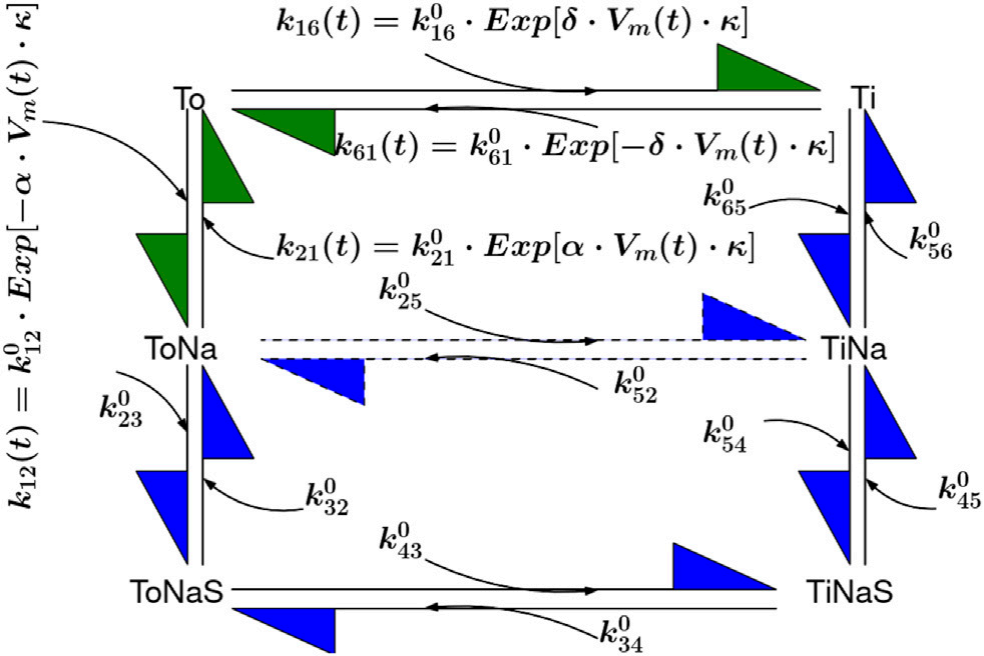
Six State Model of SGLT1. The transitions To to Ti and To to ToNa are voltage dependent and indicated with green arrows. The reactions denoted by blue arrows are voltage independent and described with constant rates $k_{xy}^{0}$ were xy describes the reaction number in the figure. δ and α are phenomenological constants with 0.7 and 0.3 and κ=F/RT , describe the fraction of the electrical field sensed by the reaction (Parent et al., 1992; Wright et al., 2011). The rate constants are listed in Table 3.

We previously showed by utilizing proteoliposomes that SGLT1 is an efficient facilitator of passive water transport (Erokhova et al., 2016). The measured unitary permeability for water amounted to 3.3 ± 0.4 × 10^−13^ cm^3^/s at 5°C. These rates were comparable to those reported for aquaporins (Erokhova et al., 2016). Water flux through SGLT1 did not depend on the presence of the co-substrate Na^+^, because it remained the same when Na^+^ was substituted with K^+^. Less water flew through SGLT1 when 20 mM glucose, a saturating concentration, was present in the bath solution. However, the reduction in the conductivity for water was small (i.e., 17%). This suggested that during transport, SGLT1 dwelled longer in states, which conducted water than in states, which were water-impermeable. Our model predicted that at the condition at which the water flux was found reduced, SGLT1 dwelled 27 percent of the time in glucose-bound states. This tempted us to speculate that the water-impermeable states were those, which were bound to glucose. This view is in line with results obtained from MD simulations of the bacterial homolog vSGLT in the inward-facing state. These showed that residues located close to the glucose binding site gated water flux (Li et al., 2013) and that the permeability for water was modulated albeit not tightly coupled to substrate release (Adelman et al., 2014). However, a shortcoming of our modeling approach is that for technical reasons water flux through SGLT1 had to be measured at 5°C, while the employed six state model (see Figure 8) was parametrized using data obtained at room-temperature. Arguably, the conformational equilibrium of SGLT1 at 5°C can differ from that at room temperature. However, the low activation energy for water transport through SGLT1, reported by others (i.e., in the range of 4.2–5 kcal/mol) (Meinild et al., 1998; Zeuthen et al., 2016), suggests that temperature dependent changes of the conformational equilibrium of the transporter are likely modest. The explanation for this could be as follows: the two reactions in the transport cycle, which ought to be most affected by temperature are the conformational rearrangements (i.e., 1) the transition, which carries substrate and co-substrate through the membrane and 2) the return of the empty transporter to the outward facing conformation). Because, the reaction paths of these two transitions are similar, there is no reason to believe that their temperature dependencies are vastly different. If true, the rates of these two reactions are expected to change with temperature by a similar factor. As a consequence of this, the conformational equilibrium (but not the glucose uptake rate), remains the same at different temperatures.

Our vesicle model is unique in that it incorporates a solute carrier. Yet, other vesicle models exist, which account for passive diffusion of water (Wachlmayr et al., 2022), weak acids (Hannesschlaeger et al., 2019a; Gabba and Poolman, 2020), and ions (Megens et al., 2014; Shen et al., 2020) through the vesicular membrane. However, due to their different scopes, none of them simultaneously modeled 1) membrane potential (Gabba and Poolman, 2020), 2) partial volume effects (Hannesschlaeger et al., 2019b; Gabba and Poolman, 2020; Shen et al., 2020), 3) de/protonation of the buffer (Gabba and Poolman, 2020; Shen et al., 2020), and 4) self-dissociation of water (Gabba and Poolman, 2020). While we modeled all of the above, we disregarded the size distribution of the vesicle ensemble as was included in models, which dealt with water (Wachlmayr et al., 2022) and weak acid permeation (Gabba and Poolman, 2020).

It is important to point out, however, that the predictive power of any given model hinges on its accurate parameterization. For example, we showed that the time course and extent by which V_M_ changed, depended 1) on the initial intra- and extra-vesicular ion concentrations and 2) on the P_M_ values used in the simulation. The rates of passive ion fluxes can be measured in experiments. However, we found for the same ion vastly different values in the literature (Table 1). The variabilities in the reported P_M_ values for the same ion likely reflect differences in the experimental conditions at which this parameter was determined (e.g., the lipid composition of the membranes), and inaccuracies of the methods used to estimate it. We, therefore, surmise that accurate parametrization of the model may necessitate measuring these rates for membranes of exactly the same lipid composition as used in the experiment. For lack of such a coherent set of values we selected for most simulations, literature values, which adhered to the generally accepted rank order of permeability: $P_{H^{+}}>P_{Cl^{-}}>P_{K^{+}}>P_{Na^{+}}>P_{NMDG^{+}}$ . In this context, it is worth mentioning that the main determinants of ion permeation are the Born penalty, the image energy, the dipole potential, and the surface potential. The Born penalty incurs on transfer of an ion from one dielectric medium (*ε*_water_ ∼ 80) into another (*ε*_membrane_ ∼ 2) (Born, 1920). It is independent of the sign of the charged molecule, decreases with increasing Born radius and is slightly lowered by a polarizing effect, which emanates from charged solutes in the vicinity of dielectric interfaces (image energy) (Parsegian, 1969). The Born-image energy decreases with decreasing membrane width. The dipole potential, on the other hand, is generated by oriented water molecules and the polar carbonyl groups of ester bonds (Gawrisch et al., 1992). Because the dipole potential is positive, it favours partitioning of anions over cations. Surface charges, selectively attract or repel anions and cations, which offsets their chemical potential (McLaughlin, 1989).

Proteoliposomes are well suited for studies of solute carrier function. This is because 1) the experimental outcomes are not confounded by the presence of other proteins, 2) the number of protein units reconstituted into a vesicle can be titrated and- if so wished- even down to a single molecule 3) the ion concentrations in and outside the vesicle can be set as desired and 4) the lipid composition of the vesicular membrane is in the hands of the experimenter. However, one major challenge when using proteoliposomes is that vesicle parameters such as membrane voltage and the intraluminal ion-concentrations are difficult to control. Here we tried to address this problem with our model. The idea was that if we cannot control these parameters, we can at the very least try to model how they change over time and on (experimental) perturbation. We demonstrated in an example that the presence of a K^+^ gradient accelerated the rate of glucose uptake and increased the concentrative power of SGLT1, although K^+^ binding was not specified in the reaction scheme of this transporter. In the model this had occurred because SGLT1 is an electrogenic transporter, which reacted to the more negative V_M_ that arose in the presence of the K^+^ gradient. In this context, it is worth mentioning that a study exists, in which it was proposed that LeuT (a bacterial homolog of neurotransmitter transporters) antiports K^+^ (Billesbolle et al., 2016). The main argument was that the concentrative power of reconstituted LeuT was found increased in the presence of a K^+^ gradient. While it may turn out that LeuT indeed antiports K^+^, this conclusion can be challenged on the grounds that the vesicle parameters, in particular V_M_ were not monitored in this study. The same is true for a more recent study on the *drosophila* dopamine transporter, which was conducted by the same group (Loland et al., 2022).

Another challenge when using proteoliposomes, is the difficulty to influence the orientation of the transporter in the membrane during reconstitution. Whereas protein orientation in cellular membranes is mainly determined by the positive-inside rule (von Heijne, 1992), with basic residues being more abundant on the cytoplasmic side, *in-vitro* protein orientation is- in most instances- random (i.e., 50% in the proper orientation and 50% inserted upside down). We accounted for this in our model by implementing both orientations with the two fractions kept tunable. This allows for predicting the impact of random-, biased- or perfect-orientation of membrane transporters on experimental outcomes. Thereby, the model can help to direct efforts to tune the orientation of reconstituted membrane transporters.

The ultimate goal of our modelling exercise is to find a model, which can faithfully predict the time dependence of the vesicle parameters. However, it is unlikely that our model already fulfills this criterion. To improve the model, its predictions must be tested in experiments. A readily accessible parameter, for instance, is the time course of volume change *via* scattering or fluorescence self-quenching experiments (Wachlmayr et al., 2022). The latter is performed *via* intra luminal trapped fluorophores as volume readout. The time course and extent of the change in the concentration of the individual ion species and the magnitude of the transmembrane potential are accessible *via* ion-(Feroz et al., 2021) or voltage- (Cortes et al., 2018; Gest et al., 2021) sensitive fluorophores. These parameters can be measured for different intra and extraluminal ion concentrations in the presence and absence of SGLT1, with and without substrate. The results of these measurements can then be compared with the predictions of the model and discrepancies therein resolved by adapting the model. However, testing the feasibility of this approach is beyond the scope of the present study, but we intend to test utility in follow-up investigations.

## 4 Materials and Methods

We simulated substrate uptake by SGLT1 embedded into a unilamellar vesicle. We accounted for solute flux through SGLT1 by employing a recently published kinetic model of this transporter (Parent et al., 1992; Adelman et al., 2016; Wright et al., 2011). Passive diffusion of solutes through the membrane was described using the Goldman-Hodgkin-Katz flux equation. If for a solute no experimentally determined P_M_ value was available, we calculated it from estimated hexadecane/water partition coefficients. The system of differential equations constituting the model, was solved numerically with Mathematica’s NDSolve IDA package (V. 12) (RRID:SCR_014448).

### 4.1 Vesicle Model

The change in vesicle volume caused by the presence of an osmotic gradient $\delta_{OSm}$ was described by the following equation:

$$\frac{dV}{dt}=S\cdot P_{f}\cdot v_{w}\cdot \delta_{OSm}+\sum_{i=0}^{k}\frac{dn_{i+}}{dt}\cdot v_{i}$$ (1)

where S , $P_{f}$ and $v_{w}$ are the surface area of the vesicle, membrane permeability for water and the partial molar volume of water, respectively. $\delta_{Osm}$ is the difference in the osmotic concentration between the inner and outer solution. The first term in Eq. 1 accounts for the volume change evoked by the osmotic gradient $\delta_{Osm}$ . The second term considers partial volume effects (Borsarelli and Braslavsky, 1998; Fucaloro et al., 2007; Millero, 2002). k is the number of solutes present in the assay. The change in the amount of each solute $\frac{dn_{i+}}{dt}$ was multiplied with its molar volume $\left(v_{i}\right)$ . The values for $v_{i}$ are listed in Table 2.

**TABLE 2:** Model parameters.

Parameter	Symbol	value	References
Vesicle radius t=0	$r_{0}$	60 nm	Horner et al. (2015)
Specific membrane capacity	μ	$1\mu F/cm^{2}$	Hannesschlaeger, Barta, et al. (2019b)
Bilayer thickness	h	3.75 nm	Paula et al. (1996)
Partial molar volume $H_{2}O$	$v_{w}$	0.018 1/mol	Hannesschlaeger, Barta, et al. (2019a)
Partial molar volume $Na^{+}$	$v_{Na}$	$-1.21\cdot 10^{-6}m^{3}/mol$	Millero, (2002)
Partial molar volume $K^{+}$	$v_{K}$	$9.02\cdot 10^{-6}m^{3}/mol$	Millero, (2002)
Partial molar volume $Cl^{-}$	$v_{Cl}$	$1.78\cdot 10^{-5}m^{3}/mol$	Millero, (2002)
Partial molar volume $OH^{-}$	$v_{OH}$	$-4.04\cdot 10^{-6}m^{3}/mol$	Millero, (2002)
Partial molar volume Glucose	$v_{Glu}$	$1.12\cdot 10^{-4}m^{3}/mol$	Fucaloro et al. (2007)
Partial molar volume $H^{+}$	$v_{H}$	$-5.5\cdot 10^{-9}m^{3}/mol$	Borsarelli & Braslavsky, (1998)
Water dissociation rate	$k_{w}^{+}$	$2.5\cdot 10^{-5}{\text{s}}^{-1}$	Stillinger, (1978)
passive $H^{+}$ permeability	$P_{H^{+}}$	$3.5\cdot 10^{-7}m/s$	Hannesschlaeger, Barta, et al. (2019b)
passive $OH^{-}$ permeability	$P_{OH^{-}}$	0m/s ^a^	
passive $Na^{+}$ permeability	$P_{Na^{+}}$	$1.5\cdot 10^{-13}m/s$	Brunner et al. (1980)
passive Glucose permeability	$P_{Glu}$	$3.1\cdot 10^{-13}m/s$	Brunner et al. (1980)
passive Sucrose permeability	$P_{Suc}$	$3.1\cdot 10^{-14}m/s$	Stefely et al. (2002)
passive $K^{+}$ permeability	$P_{K^{+}}$	$3.\cdot 10^{-13}m/s$	Paula et al. (1996)
passive $Na^{+}$ permeability	$P_{Cl^{-}}$	$5.\cdot 10^{-13}m/s$	Toyoshima & Thompson, (1975)
HEPES		0m/s ^b^	
NMDG^+^	$P_{NMDG}$ +	$1.6\cdot 10^{-21}m/s$	prediction
NMDG_0_	$P_{\text{NMDG}0}$	$\left(1.6\cdot 10^{-21}-1.\cdot 10^{-11}\right)m/s$ ^c^	Experimental, prediction, (Reuss, 1979; Dhoke et al., 2005; Elustondo et al., 2013)

a We set $P_{OH^{-}}$ to zero as at pH 7.5 with equal concentrations of $H^{+}$ and $OH^{-}$ $P_{OH^{-}}$ , can be neglected. $P_{H^{+}}$ exceeds $P_{OH^{-}}$ by more than two orders of magnitude (Gutknecht and Walter, 1981b).

b The contribution of HEPES, is negligible due to its low concentration and low predicted membrane permeabilities. Moreover, exact treatment is challenging due to its zwitterionic states.

c In Figure 3 we showed that the potential effect of a P_NMDG__0_ permeability of $1.0\cdot 10^{-11}\frac{m}{s}$ is minor. Therefore, and due to the lack of more accurate values we used $P_{NMDG^{+}}=P_{NMDG_{0}}$ elsewhere.

**TABLE 3:** The six different states after Figure 8 and corresponding shortcuts used in the code implementation are listed in the first two columns. The overall rate gain and loss is always contributed to the corresponding state in the row. The right column refers to the initial rates given at t=0 after Table S3 in Adelman et al. (2016). The remaining rates $k_{54}^{0\text{*}}$ and $k_{52}^{0\text{*}}$ were estimated according to Kirchhoff’s mesh rule by multiplying all rates along the states To—ToNa—ToNaS—TiNaS—TiNa—Ti—To and subtracting them from the other direction by setting the difference to zero. For $k_{52}^{0\text{*}}$ the mesh ToNa—ToNaS—TiNaS—TiNa—ToNa was used for evaluation. Values are given for 22°C.

State	Abbreviation	gain	Loss	Corresponding $k^{0}$ values [1]
apo outward facing state	To(t)	$k_{61}\left(t\right)+k_{21}\left(t\right)$	$k_{16}\left(t\right)+k_{12}\left(t\right)$	$k_{61}^{0}=251/s$ , $k_{16}^{0}=6001/s$ , $k_{12}^{0}=500001/M^{2}s$ , $k_{21}^{0}=3001/s$
apo inward facing state	Ti(t)	$k_{16}\left(t\right)+k_{54}^{0}$	$k_{61}\left(t\right)+k_{45}^{0}$	$k_{61}^{0}=251/s$ , $k_{16}^{0}=6001/s$ , $k_{54}^{0\text{*}}≃444441/M$ , $k_{45}^{0}=8001/s$
sodium bound outward facing state	ToNa(t)	$k_{12}\left(t\right)+k_{52}^{0}+k_{32}^{0}+k_{72}^{0}$	$k_{21}\left(t\right)+k_{25}^{0}+k_{23}^{0}+k_{27}^{0}$	$k_{12}^{0}=500001/M^{2}s$ , $k_{52}^{0\text{*}}≃0.0002469141/s$ , $k_{25}^{0}=0.011/s$ , $k_{32}^{0}=201/s$ , $k_{23}^{0}=450001/Ms$ , $k_{72}^{0}=0.011/s$ , $k_{27}^{0}=500001/s$
sodium bound inward facing state	TiNa(t)	$k_{65}^{0}+k_{25}^{0}+k_{45}^{0}$	$k_{56}^{0}+k_{52}^{0}+k_{54}^{0}$	$k_{65}^{0}=45001/M^{2}s$ , $k_{56}^{0}=161/s$ , $k_{52}^{0\text{*}}≃0.000246914\;1/s$ , $k_{25}^{0}=0.011/s$ , $k_{54}^{0\text{*}}≃444441/\mathrm{Ms}$ , $k_{45}^{0}=8001/s$
sodium and glucose bound outward facing state	ToNaS(t)	$k_{23}^{0}+k_{34}^{0}$	$k_{32}^{0}+k_{43}^{0}$	$k_{32}^{0}=201/s$ , $k_{23}^{0}=450001/Ms$ , $k_{34}^{0}=501/s$ , $k_{43}^{0}=501/s$ ,
sodium and glucose bound inward facing state	TiNaS(t)	$k_{43}^{0}+k_{54}^{0}$	$k_{34}^{0}+k_{45}^{0}$	$k_{34}^{0}=501/s$ , $k_{43}^{0}=501/s$ , $k_{54}^{0\text{*}}≃444441/\mathrm{Ms}$ , $k_{45}^{0}=8001/s$

$\delta_{OSm}$ was calculated as follows:

$$\delta_{Osm}=\left(\sum_{i=0}^{k}{\left[X\left(t\right)\right]}_{i}^{inside}+\sum_{i=0}^{n}{\left[Y\left(t=0\right)\right]}_{i}^{inside}\cdot \frac{V_{0}}{V\left(t\right)}\right)-\left(\sum_{i=0}^{m}{\left[Z\left(t\right)\right]}_{i}^{outside}\right)$$ (2)

where ${\left[X\left(t\right)\right]}^{inside}$ is the luminal concentration of the (k) permeable solutes and ${\left[Y\left(t=0\right)\right]}^{inside}$ the luminal concentration of the (*n*) impermeable solutes. ${\left[Z\left(t\right)\right]}^{outside}$ is the sum of all extra-vesicular solute concentrations (m). $V_{0}$ is the initial volume of the lipid vesicle (Table 2):

$$V_{0}=\frac{4}{3}\pi \cdot {\left(r_{0}-h\right)}^{3}$$ (3)

where we corrected the outer radius $r_{0}$ with the membrane bilayer thickness h.

We used the Goldman-Hodgkin-Katz flux equation to describe passive diffusion of solutes through the membrane

$$j_{GHK}\left(t\right)=\frac{dn_{q}}{dt}=-\frac{z_{q}\cdot S\cdot V_{M}\left(t\right)\cdot P_{M,q}\cdot F}{R\cdot T}\cdot \frac{{\left[q\right]}_{in}-{\left[q\right]}_{out}\cdot Exp\left[-\frac{z_{q}\cdot V_{M}\left(t\right)\cdot F}{R\cdot T}\right]}{1-Exp\left[-\frac{z_{q}\cdot V_{M}\left(t\right)\cdot F}{R\cdot T}\right]}$$ (4)

where $P_{\text{M},\text{q}}$ , $\;z_{q}$ , F , R , ${\left[\text{q}\right]}_{in}$ , and ${\left[q\right]}_{out}$ are the membrane permeability of species q, its valance, the Faraday constant, the Gas constant and the concentration of the charged species q inside and outside the vesicle, respectively. In the simulation, the charged species were $Na^{+}$ , $K^{+}$ , $H^{+}$ , $NMDG^{+}$ , and $Cl^{-}.$ $V_{M}$ is the membrane potential, which at time t=0 was set to zero. $P_{\text{M},\text{q}}$ values used in the simulations are listed in Table 2.

The membrane was modeled as a capacitor, which was charged by the charged molecules entering the vesicle. For membrane potentials below $V_{M}<300$ mV (Benz et al., 1979) electrical breakdowns of the lipid bilayer can be neglected and the vesicle membrane can be described as a capacitor with charge difference Q: (Hannesschlaeger et al., 2019a; Montal and Mueller, 1972)

$$Q=\mu \cdot S\cdot V_{M}$$ (5)

where μ is the specific membrane capacity and *S* the surface area.

Q can be expressed as: $Q=n_{q}\cdot z_{q}\cdot F$ . From this we can obtain $V_{M}$ :

$$V_{M}=-\frac{\sum_{q=0}^{k}\left[n_{q}\cdot z_{q}\right]\cdot F}{S\cdot \mu }$$ (6)

where q indicates a charged species that can diffuse through the membrane with its corresponding amount $n_{q}$ and valence $z_{q}$ . The impermeable solutes do not contribute to $V_{M}$ .

Since permeation of free ions like $H^{+}$ and $OH^{-}$ also contributes to $V_{M}$ we considered also self-dissociation of water into protons and hydroxide ions according to (Hannesschlaeger et al., 2019b):

$$\frac{d\left[OH^{-}\right]}{dt}=\frac{d\left[H^{+}\right]}{dt}=k_{w}^{+}\cdot \left[H_{2}O\right]-k_{w}^{-}\cdot \left[OH^{-}\right]\cdot \left[H^{+}\right]$$ (7)

where $k_{w}^{+}$ and $k_{w}^{-}=\left[H_{2}O\right]\cdot k_{w}^{+}/K_{w}$ are the dissociation and the association rates of water with $\left[H_{2}O\right]=v_{w}^{-1}$ and $K_{w}$ the water equilibrium constant. Additionally to the change in pH described in Eq. 7, the proton concentration $\left[H^{+}\right]$ depends on the protonation and deprotonation rates of the available buffer M, the concentration of the deprotonated $\left[M^{-}\right]$ and protonated buffer form [MH] inside the vesicle can be calculated using the following relations between the protonated and deprotonated species of buffer M:

$$\frac{d\left[M^{-}\right]}{dt}=\frac{1}{V\left(t\right)}\cdot \frac{dn_{M}-}{dt}=k_{d}\cdot \left[MH\right]-k_{p}\cdot \left[H^{+}\right]\cdot \left[M^{-}\right]$$ (8)

$$\frac{d\left[MH\right]}{dt}=\frac{1}{V\left(t\right)}\cdot \frac{dn_{MH}}{dt}=-k_{d}\cdot \left[MH\right]+k_{p}\cdot \left[H^{+}\right]\cdot \left[M^{-}\right]$$ (9)

For the formulation of Eq. 8 and Eq. 9 we used the following relation: c=n/V(t) , where *c* is the concentration, V(t) the vesicle volume and n the amount of solute. The corresponding protonation rates $k_{p}$ and deprotonation rates $k_{d}$ can be estimated from a linear regression of Figure 4 in (Gutman and Nachliel, 1990):

$$\begin{array}{ccc} k_{d}=1.\cdot 10^{-0.98+pK\cdot 10.98} & k_{p}=\frac{k_{d}}{K_{A}} & K_{A}=1.\cdot 10^{-pK} \end{array}$$ (10)

where pK is the pK value of the buffer M and $K_{A}$ the corresponding equilibrium constant. To calculate the change in free protons $dn_{H^{+}}/dt$ inside the vesicle according to Eq. 4, every reaction involving proton exchange as in Eqs 7–9 was considered in the form of:

$$\frac{dn_{H^{+}}}{dt}=j_{GHK}^{H^{+}}+\left(k_{w}^{+}\cdot \left[H_{2}O\right]-k_{w}^{-}\cdot \left[OH^{-}\right]\cdot \left[H^{+}\right]+\left(\frac{d\left[M^{-}\right]}{dt}\right)\right)\cdot V\left(t\right)$$ (11)

We predicted water flux through proteoliposome by emulating stopped flow experiments. For these it was necessary to change the osmotic concentration outside the vesicle by adding an osmolyte such as glucose or sucrose with a concentration $\left[S_{add}\right]$ . Addition occurred at time $t_{s}$ and was modeled as an exponential rise (Eq. 12) with a rate constant of $k_{0}=300s^{-1}$ to account for the dead time of the stopped flow mixing process:

$$\frac{d\left[S_{tot}\right]}{dt}=\{\begin{array}{c} 0,t<t_{s}\\ k_{0}\cdot \left[S_{add}\right]-k_{0}\cdot \left[S_{tot}\right],t\geq t_{s} \end{array}$$ (12)

The total concentration can be calculated as: $\left[S_{tot}\right]\left(t\right)=\left[S\right]\left(t\right)+\left[S_{0}\right]$ , where $\left[S_{0}\right]$ is the concentration at the start of the application.

### 4.2 SGLT1 Model

**FIGURE 9:**
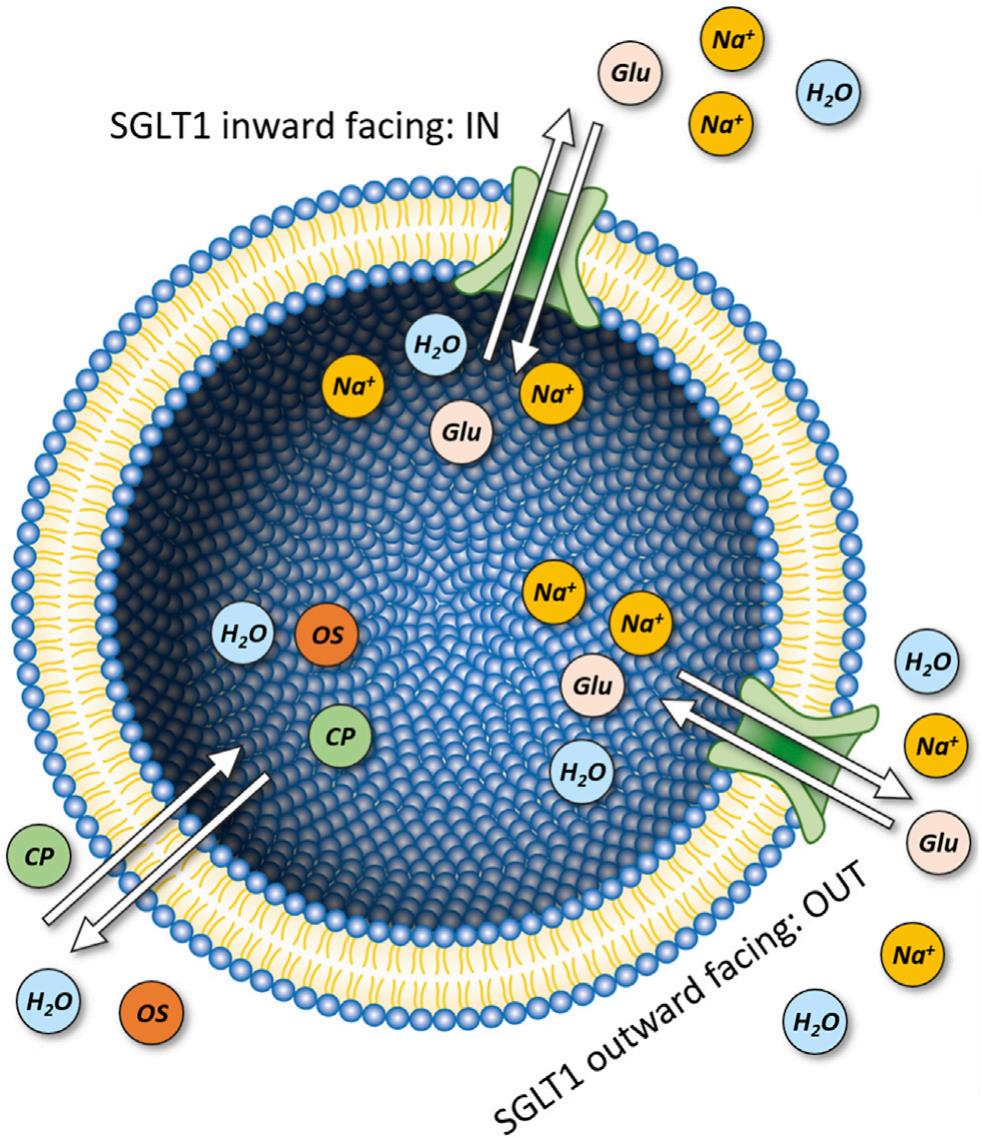
Vesicles containing SGLT1. Model of a unilamellar vesicle with SGLT1 incorporated in two different orientations. The inward- and outward facing configurations oriented towards the intra and extra luminal side are denoted as IN and OUT, respectively. In each transport cycle SGLT1 transports 2 Na^+^ ions and one glucose molecule. The model takes into account passive permeation of osmotically active solutes (OS) (i.e., sucrose, glucose, and NMDG_0_) and charged particles (CP) (i.e., Na^+^, K^+^, NMDG^+^, Cl^−^, and protons) as well as passive flux of water.

SGLT1 was modeled utilizing a recently published *Six State Model* (see Figure 8) (Adelman et al., 2016; Parent et al., 1992; Wright et al., 2011). Voltage dependent transitions are indicated with green arrows, all other transitions with blue arrows. The published model accounts for Na^+^ slippage (dotted arrow between TiNa(t) and ToNa(t) ). The transition rates used in the model were adapted from Table S3 in Adelman et al. (2016) and they are listed in Table 3. When we incorporated the kinetic model of SGLT1 into the vesicle we also accounted for: 1) transporter orientation (*IN* and *OUT*) after insertion into the vesicular membrane (see Figure 9) and 2) for passive water flux through the transporter. We implemented the two orientations of the transporter by switching in the equations, which constitute the kinetic model the concentration terms (i.e., [Na]_OUT_ and [glucose] _OUT_ became [Na]_IN_ and [glucose] _IN_ and vice versa) (Parent et al., 1992). The outward facing conformation of the fraction of the properly oriented transporters interacted with [Na] _OUT_ and [glucose] _OUT_. For the fraction which was inserted upside down it was opposite.

We introduced a passive water permeability for SGLT1 according to Erokhova et al. (2016) in our model. This enhanced the rate of volume shrinkage according to Eq. 1. As a first estimate we assumed that SGLT1 is only water permeable in its apo states but not in the glucose bound states. Therefore, we weighted the water permeability $P_{f}$ of SGLT1 with the corresponding state occupancies:

$$\begin{array}{l} P_{f}=P_{f,fast}\cdot (To_{OUT}\left(t\right)+Ti_{OUT}\left(t\right)+ToNa_{OUT}\left(t\right)+TiNa_{OUT}\left(t\right)+To_{IN}\left(t\right)\\ +Ti_{IN}\left(t\right)+ToNa_{IN}\left(t\right)+TiNa_{IN}\left(t\right))+P_{f,passive}, \end{array}$$ (13)

The passive water permeability through the membrane is: $P_{f,passive}=20\mu m/s$ (Pohl et al., 2001). The fast water permeability $P_{f,fast}$ is the estimated single channel water permeability $p_{f}=5\cdot 10^{-19}m^{3}/s$ at $22^{\circ }C$ assuming an activation energy of ∼4kcal/mol (Erokhova et al., 2016):

$$P_{f,fast}=N\cdot \frac{p_{f}}{4\pi r_{0}^{2}}$$ (14)

where N is the number of SGLT1 transporters per vesicle.

We calculated the membrane potential $V_{M}\left(t\right)$ according to Eq. 6. Sodium uptake into the vesicle ( ${Na}_{up}^{+}\left(t\right)$ in mol) by SGLT1 was computed for the different orientations as:

$$\begin{array}{l} \frac{{dNa}_{up}^{+}}{dt}=2\cdot (-\left(To_{IN}\left(t\right)\cdot k_{12}\left(t\right)\cdot {\left[Na_{in}^{+}\right]}^{2}-ToNa_{IN}\left(t\right)\cdot k_{21}\left(t\right)\right)\cdot \left(N\cdot \frac{p_{IN}}{N_{A}}\right)\\ \;\;\;\;\;\;\;\;\;\;\;\;\;\;+\left(TiNa_{OUT}\left(t\right)\cdot k_{21}\left(t\right)-Ti_{OUT}\left(t\right)\cdot k_{12}\left(t\right)\cdot {\left[Na_{in}^{+}\right]}^{2}\right)\cdot \left(N\cdot \frac{p_{OUT}}{N_{A}}\right)) \end{array}$$ (15)

The factor two at the beginning of Eq. 15 accounts for the fact that in each transport cycle two sodium ions are transported. $N_{A}$ is *Avogadro’s constant*, $p_{in}$ and $p_{out}$ are the fractions of the two possible orientations of SGLT1 after integration into the membrane ( $0\leq p_{OUT}+p_{IN}\leq 1$ ). The total sum of outward and inward configured occupancies is $p_{OUT}$ and $p_{IN}$ , respectively. The total contribution of sodium $dn_{{Na}^{+}}/dt\left[mol/s\right]$ to the estimated $V_{m}\left(t\right)$ is the sum of Eq. 15 and Eq. 4:

$$\begin{array}{l} \frac{{dn}_{{Na}^{+}}}{dt}=\frac{{dNa}_{up}^{+}}{dt}+j_{GHK}^{Na^{+}}\\ \;\;\;\;\;\;\;\;\;\;\;=\frac{{dNa}_{up}^{+}}{dt}-\frac{z_{{\text{Na}}^{+}}\cdot S\cdot V_{m}\left(t\right)\cdot P_{Na^{+}}\cdot F}{R\cdot T}\cdot \frac{{\left[Na^{+}\right]}_{in}-{\left[Na^{+}\right]}_{out}\cdot Exp\left[-\frac{z_{{\text{Na}}^{+}}V_{M}\left(t\right)F}{R\cdot T}\right]}{1-Exp\left[-\frac{z_{{\text{Na}}^{+}}V_{M}\left(t\right)F}{R\cdot T}\right]} \end{array}$$ (16)

where S , V(t) , $P_{{\text{Na}}^{+}}$ , $\;z_{{\text{Na}}^{+}}$ , F , R , T , ${\left[{\text{Na}}^{+}\right]}_{in}$ , ${\left[{\text{Na}}^{+}\right]}_{out}$ are the vesicle surface, the membrane potential, the passive ${\text{Na}}^{+}$ membrane permeability, its ion valence, the Faraday constant, the temperature, the Gas constant and the concentration of ${\text{Na}}^{+}$ inside and outside the vesicle, respectively.

Glucose uptake $S_{up}\left(t\right)$ [mol] through SGLT1 into the vesicle is:

$$\begin{array}{l} \frac{dS_{up}}{dt}=\left(-\left(ToNa_{IN}\left(t\right)\cdot k_{23}^{0}\cdot {\left[S\right]}_{in}\left(t\right)-ToNaS_{IN}\left(t\right)\cdot k_{32}^{0}\left(t\right)\right)\cdot N\cdot \frac{p_{IN}}{N_{A}}\right)\\ \;\;\;\;\;\;\;\;\;\;\;+\left(\left(TiNaS_{OUT}\left(t\right)\cdot k_{45}^{0}-TiNa_{OUT}\left(t\right)\cdot k_{54}^{0}\cdot {\left[S\right]}_{in}\left(t\right)\right)\cdot N\cdot \frac{p_{OUT}}{N_{A}}\right) \end{array}$$ (17)

Since glucose is also an osmolyte but uncharged, the passive glucose flux $j_{s}\left(t\right)$ through the membrane was taken into account by using *Fick’s first diffusion Law* (Wang et al., 2014):

$$j_{s}\left(t\right)=-\left(S\cdot P_{Glu}\cdot \left({\left[S\right]}_{in}\left(t\right)-{\left[S\right]}_{out}\left(t\right)\right)\right)$$ (18)

where ${\left[S\right]}_{out}\left(t\right)$ is the extra-vesicular glucose concentration. The total glucose uptake into the vesicle is the sum of Eqs 17, 18.

### 4.3 Estimation of Passive Membrane Permeabilities

For solutes for which experimentally determined P_M_-values were unavailable, we calculated them from the estimated hexadecane/water partition coefficients K_hex/w_ in accordance with the solubility diffusion model, using semi-empirical correlations for neutral (Walter and Gutknecht, 1986), anionic (Ebert et al., 2018), and cationic (Ebert, 2020) molecules. Predicted values and the pertinent correlations are listed in Table 4. Partition coefficients were predicted using commercial software based on quantum chemical calculations and the COSMO-RS (“Conductor-like Screening Model for Realistic Solvation”) theory. Thereby, Turbomole (TURBOMOLE V7.3 2018, a development of University of Karlsruhe and Forschungszentrum Karlsruhe GmbH, 1989–2007, TURBOMOLE GmbH, since 2007; available from http://www.turbomole.com) was used to generate so called COSMOfiles—surface charge densities of energetically optimized structures. Using COSMOconf (COSMOlogic GmbH & Co. KG; http://www.cosmologic.de: Leverkusen, Germany) we accounted for different possible conformers. COSMOtherm (Release 18 [2018] COSMOlogic GmbH & Co. KG) was used to calculate the partition coefficients. For more details see (Eckert and Klamt, 2002). All values were calculated at 22°C using the BP_TZVPD_FINE_parametrization.

**TABLE 4:** Estimated values for passive HEPES_0_, HEPES^-^, glucose, sucrose, NMDG_0_, and NMDG^+^ membrane permeabilities, the corresponding hexadecane/water partition coefficients K_hex/w_ (see Sections 4.3 and 4.4) and the pertinent correlations to calculate logP.

Compound	Log. Passive permeability log *P* [m/s]	Log K_hex/w_	Used correlation	References
NMDG^+^	−20.8	−21.2	$\log\;P=0.6*\log\;K_{hex/w}-8.1$	Ebert (2020)
HEPES^-^	−17.4	−33.7	$\log\;P=0.6*\log\;K_{hex/w}+2.8$	Ebert et al. (2018)
HEPES	−20.1^a^		$\log\;P=1.06*\log\;K_{hex/w}-0.9$	(Walter and Gutknecht, 1986)
Zwitterion 1	−19.9	−17.9		
Zwitterion 2	−39.7	−35.1		
NMDG	−7.6	−6.3		
Glucose	−12.6	−11.0		
Sucrose	−17.1	−15.3		

a Total zwitterionic HEPES permeability was calculated by multiplying the fraction of zwitterion 1 with the corresponding permeability. The HEPES zwitterion is present 59% of the time as zwitterion 1 (SMILES: OCCN1CC [NH+] (CC1)CCS([O-]) (=O) = O) and 41% as zwitterion 2 (SMILES: OCC [NH+] 1CCN (CC1) CCS ([O-]) (=O) = O) according to JChem for Office (Excel). This information was used for micro-pK_a_ calculation [JChem for Office 20.2.0.589, 2020, ChemAxon (http://www.chemaxon.com)].

### 4.4 Reliability of Predictions for Passive Membrane Permeabilities

**FIGURE 10:**
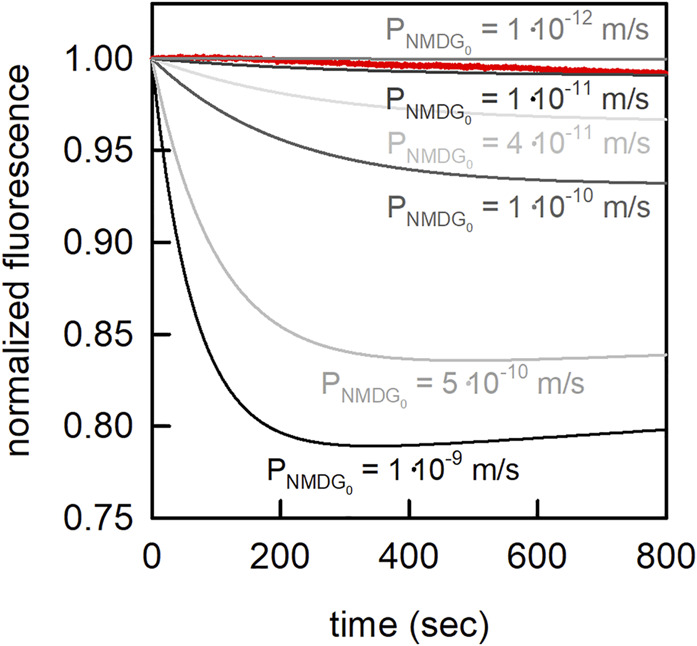
Estimation of the passive membrane permeability of NMDG_0_. In the presence of an outwardly directed NMDG gradient (10 mM inside to 3.33 mM outside) NMDG_0_ diffused out of the vesicle, which led to a drop of the intraluminal pH. The extra- and intraluminal solutions contained 1 mM MES, and 100 mM NaCl. The pH on both sides of the membrane was 7.0. Shown in red is the measured change in carboxyfluorescein fluorescence on imposition of the NMDG gradient at time point zero. From these measurements we estimated an upper limit for the membrane permeability of $NMDG_{0}∼10^{-11}m/s$

A deviation of about 1 log unit between an experimentally determined and predicted *p*-value is normal when using COSMOtherm to calculate the membrane permeability of neutral solutes (Schwobel et al., 2020). For glucose the predicted value (listed in Table 4) and the experimentally determined value were in good agreement (P_exp_ = 3.0⋅10^−13^ m/s (Brunner et al., 1980); P_pred_ = 2.5⋅10^−13^ m/s). The deviation in the case of sucrose was higher (P_exp_ = 3.1⋅10^−14^ m/s (Stefely et al., 2002); P_exp_ = 8⋅10^−16^ m/s (Brunner et al., 1980); P_pred_ = 8⋅10^−18^ m/s). NMDG can exist in a neutral and in a charged form (i.e., NMDG_0_ and NMDG^+^; pKa = 8.8). The predicted permeability of NMDG_0_ was 2.5⋅10^−8^ m/s. This value seemed high given that NMDG was thought to permeate slower through membranes than sodium or potassium ions (Dhoke et al., 2005; Elustondo et al., 2013; Reuss, 1979). To resolve this discrepancy, we determined the NMDG_0_ permeability experimentally. These measurements allowed for calculating an upper limit of $∼10^{-11}$ $\frac{m}{s}$ for $NMDG_{0}$ (Figure 10). The large deviation between predicted and measured value can be explained by conformer effects (22 relevant conformers were detected by COSMOconf, other important conformers might have been overlooked) and possible tautomeric effects. In addition, the complexity of the NMDG molecule (i.e., it harbors multiple functional groups) leads to larger prediction uncertainties (Ulrich et al., 2021). Due to the strongly decreased membrane permeability of cations as compared to their neutral counterparts, a significant NMDG^+^ membrane permeability can be excluded.

### 4.5 Experimental Estimation of the Neutral NMDG Species Membrane Permeability

We measured $NMDG_{0}$ permeability with a pH sensitive vesicle assay (Hannesschlaeger et al., 2019a). In brief, *E. coli* polar lipids (PLE, Avanti Polar Lipids) were dried on a rotary evaporator. The thin lipid film was rehydrated in 100 mM NaCl, 1 mM MES, 0.5 mM Carboxyfluorescein, and 10 mM NMDG-Cl at pH 7.0 to obtain a final lipid concentration of 10 mg/ml. Subsequently, the solution was extruded through polycarbonate filters with 100-nm pore sizes using a mini-extruder from Avanti Polar Lipids. Free Carboxyfluorescein was removed using a PD10 column. Vesicle formation was confirmed by dynamic light scattering (DELSA Nano HC, Beckmann Coulter, data not shown). Finally, the 4x diluted vesicle suspension was mixed in a 1:2 ratio at room temperature with a solution void of NMDG-Cl in a stopped-flow apparatus (µ-SFM, Bio-Logic, Claix, France). At the final extra-vesicular ion concentrations (i.e., 100 mM NaCl, 1 mM MES, and 3.3 mM NMDG-Cl at pH 7.0) $NMDG_{0}$ effluxed out of the vesicles. The resulting drop in pH inside the vesicle was monitored using carboxyfluorescein at an excitation wavelength of 480 nm and a detector angle of 90°. At least three single shots were recorded and averaged per experiment. To correct for photo bleaching of carboxyfluorescein the same experiment was repeated using 10 mM NMDG-Cl outside, to eliminate efflux of NMDG. The experiment was repeated three times. To estimate an upper limit of $NMDG_{0}$ membrane permeability we simulated the kinetics using similar conditions but $NMDG_{0}$ flux rates varying between $10^{-9}-10^{-12}m/s$ .

## Data Availability

The original contributions presented in the study are included in the article/Supplementary Material, further inquiries can be directed to the corresponding author.
